# Maternal effects in vulnerability to eye‐parasites and correlations between behavior and parasitism in juvenile Arctic charr

**DOI:** 10.1002/ece3.3428

**Published:** 2017-09-18

**Authors:** Raine Kortet, Tiina Lautala, Jukka Kekäläinen, Jouni Taskinen, Heikki Hirvonen

**Affiliations:** ^1^ Department of Environmental and Biological Sciences University of Eastern Finland Joensuu Finland; ^2^ Behaviour, Ecology and Evolution Team (Integrative Ecology Unit) Department of Biological and Environmental Sciences University of Helsinki Helsinki Finland; ^3^ Department of Biological and Environmental Science University of Jyväskylä Jyväskylä Finland

**Keywords:** antipredation behavior, *Diplostomum* eye flukes, hatchery‐raised, immunocompetence, parasite resistance, salmonid

## Abstract

Hatchery‐reared fish show high mortalities after release to the wild environment. Explanations for this include potentially predetermined genetics, behavioral, and physiological acclimation to fish farm environments, and increased vulnerability to predation and parasitism in the wild. We studied vulnerability to *Diplostomum* spp. parasites (load of eye flukes in the lenses), immune defense (relative spleen size) and antipredator behaviors (approaches toward predator odor, freezing, and swimming activity) in hatchery‐reared juvenile Arctic charr (*Salvelinus alpinus*) using a nested mating design. Fish were exposed to eye‐fluke larvae via the incoming water at the hatchery. Fish size was positively associated with parasite load, but we did not find any relationship between relative spleen size and parasitism. The offspring of different females showed significant variation in their parasite load within sires, implying a dam effect in the vulnerability to parasites. However, the family background did not have any effect on spleen size. In the mean sire level over dams, the fish from the bolder (actively swimming) families in the predator trials suffered higher loads of eye flukes than those from more cautiously behaving families. Thus, the results indicate potentially maternally inherited differences in vulnerability to eye‐fluke parasites, and that the vulnerability to parasites and behavioral activity are positively associated with each other at the sire level. This could lead to artificial and unintentional selection for increased vulnerability to both parasitism and predation if these traits are favored in fish farm environments.

## 1 INTRODUCTION

Every year, billions of farm‐raised fish are released into lakes, rivers, and seas to support, rehabilitate, and maintain natural populations (e.g., Brown & Day, 2002; McNeil, 1991; Neff, Garner, & Pitcher, 2011). However, the low survival of the introduced fish relative to wild fish is a major issue, often affecting the success of breeding programs (e.g., Neff et al., 2011). Accordingly, mortality of the introduced fish can be up to ten times higher than among fish from the wild (Brown & Day, 2002; Olla, Davis, & Ryer, 1998). The main proximate causes for mortality, in general, are biotic (e.g., predation, parasitism, and competition for resources) or abiotic (e.g., pollution, eutrophication or other factors affecting physical characteristics of the water body) (Braum, 1978). Generally, it has been shown that captive breeding and rearing causes biological changes that lower the fitness of reared fish in the wild, and the ultimate causes behind the issue are being discussed (e.g., Álvarez & Nicieza, 2003; Fleming & Petersson, 2001; Houde, Garner, & Neff, 2015; Huntingford, 2004; Kekäläinen et al., 2013; Olla et al., 1998; Saikkonen, Kekäläinen, & Piironen, 2011).

One possible explanation for the low survival of hatchery‐reared fish in the wild is a relatively low degree of genetic variation among fish farm and hatchery populations (Wedekind & Müller, 2004). Low genetic variation might quickly lead to inbreeding depression and result in a population, that is not able to respond to changes in the environment. Genetic variation in important fitness‐related traits, such as antipredator behavior and vulnerability to parasitism, may play an essential role in this. Another explanation for the low survival is connected to life history evolution. According to life history theory, organisms are expected to show trade‐offs between different life history traits (Roff, 1992; Stearns, 1992). Empirical and experimental results indicate the existence of those trade‐offs, for example, between parasite resistance and different life history traits (Kekäläinen, Pirhonen, & Taskinen, 2014a; Kortet, Vainikka, Rantala, Myntti, & Taskinen, 2004; Lochmiller & Deerenberg, 2000; Norris & Evans, 2000). Trade‐offs have been proposed to occur also between predator avoidance behavior, immune defense, and growth rate (e.g., Kortet, Hedrick, & Vainikka, 2010; Rigby & Jokela, 2000). Thus, fish farm conditions might favor and produce a ‘farm stock’ equipped with traits that are beneficial to the fish farm environment (lacking certain predators, parasites, etc.) relatively quickly at the expense of natural antipredatory behavior or decreased vulnerability to parasitism, which may lead to poor survival in the wild. Presumably, natural selection acts to favor weaker antipredator behavior in habitats with lower predation risk (Chivers & Smith, 1998; Chivers, Wildy, Kiesecker, & Blaustein, 2001; Lima & Dill, 1990; Riechert & Hedrick, 1990) and this is, in general, bound to the co‐evolutionary cycles between predator and prey (cf. “Red Queen” hypothesis of Van Valen, 1973). Supporting this possibility, experimental studies have clearly demonstrated that predation can have an effect on various fitness‐related traits (e.g., Grostal & Dicke, 1999; Hedrick & Kortet, 2006). If certain inherited behavioral traits, like high swimming activity associated with increased feeding activity, are favored in the fish farm populations (see Huntingford, 2004), it can also reflect on other traits, like vulnerability to parasitism. Moreover, the selective pressure by certain parasites might be weaker in farms compared with wild environment as farm environment is more favorable for hosts as means of increased nutrition and decreased or nonexistent predation. This may be especially true for parasites in the intermediate stages of their life cycle, as in the farm environment these parasites face ecological dead ends when their transmission to the definite piscivorous hosts is prevented. In the wild, these parasites may cause increased risk to predation by potentially manipulating the intermediate host behavior (e.g., Gopko, Mikheev, & Taskinen, 2017; Kekäläinen, Lai, Vainikka, Sirkka, & Kortet, 2014b; Seppälä, Karvonen, & Valtonen, 2006; but see Klemme, Kortet, & Karvonen, 2016). Moreover, recently it was shown that in the fish farms also rearing practices can affect parasitism and host parasite resistance (Karvonen et al., 2016).

A trade‐off between immunological defense and life history traits has been suggested to be particularly important (Lochmiller & Deerenberg, 2000; Norris & Evans, 2000; Viney, Riley, & Buchanan, 2005). The host immune system fights against parasites and diseases to reduce the fitness costs of parasitism (Goater & Holmes, 1997), but at the same time the defense is traded off for other energy demanding functions, like self‐maintenance. Thus, if antipredation behavior limits an individual's access and competition for nutritional resources, consequences should be seen in its capability to respond immunologically against parasites and pathogens. There are only a few studies examining potential immunologic costs of the antipredation behavior (e.g., Kortet, Rantala, & Hedrick, 2007; Kortet et al., 2010 Rigby & Jokela, 2000). Rigby and Jokela (2000) experimentally demonstrated that increased levels of predation‐avoidance behavior in freshwater snail (*Lymnea stagnalis*), reduced the snail's ability to defend against potential pathogens. In generally, if this type of predator‐mediated negative resource‐mediated response in individual's immunocompetence exists, even a short scale increase in predation could mean increased risk to get infected by parasites and pathogens, and lowered tolerance of the already existing infections (Rigby & Jokela, 2000). However, genetic associations between vulnerability to parasitism and antipredation behaviors have received less attention (Kortet et al., 2010).

Diplostomatidae eye flukes are highly prevalent parasites in numerous wild fishes (e.g., Valtonen & Gibson, 1997). *Diplostomum* trematode parasites are common also in fish farms around the world, and they are found in several commercial salmonid species used in fish industry. *D. spathaceum* parasites cause impaired vision and even blindness for fish by forming cataracts (Kuukka‐Anttila, Peuhkuri, Kolari, Paananen, & Kause, 2010). This has been suggested as a notable factor affecting survival of fish by increasing risk of predation and lowering feeding abilities depending on the environment (e.g., Gopko et al., 2017; Mikheev, Pasternak, Taskinen, & Valtonen, 2010; Seppälä, Karvonen, & Valtonen, 2004). *Diplostomum* larvae can affect the behaviors of their hosts as well (Gopko, Mikheev, & Taskinen, 2015; Mikheev et al., 2010). However, host fish have been shown to develop some acquired resistance against *D. spathaceum* (Kalbe & Kurtz, 2006; Karvonen, Hudson, Seppälä, & Valtonen, 2004a), but it is not known whether vulnerability against *Diplostomum* infections in salmonids has a heritable component (but see Kuukka‐Anttila et al., 2010). On the other hand, Voutilainen et al. (2009) demonstrated geographical variation in infectivity of *Diplostomum* on salmonids.

In this study, we examined vulnerability of naïve juvenile fish to *Diplostomum* spp. eye flukes (parasite load after natural controlled infection) and immune defense (relative spleen size) and antipredator behavior in hatchery‐reared juvenile Arctic charr (*Salvelinus alpinus*) using a nested mating design. The current charr population had been held in a hatchery environment for two generations due to its endangered status. In this breeding program, older female fish as dams are considered particularly valuable and are often used multiple times. We were interested whether offspring from different dams would vary in their vulnerability to parasitism. Our ultimate aim was to reveal potentially heritable variation in vulnerability to *Diplostomum* parasites, and study whether vulnerability to parasites is associated with behavioral traits at the mean sire level over dams. To reach our aim, we first raised 28 families of Arctic charr in two replicates, and then measured various behavioral traits as potential antipredation responses from the juvenile fish of these same families. Finally, we measured *Diplostomum* eye‐parasite loads and relative spleen sizes of the fish. In addition, we studied the sire‐level correlations over dams between fish vulnerability to *Diplostomum* parasitism and antipredator behavior. Heritable variation in behavioral traits per se will be reported elsewhere (T. Lautala, unpublished data).

## 2 MATERIALS AND METHODS

### 2.1 Study animals, fertilizations, and hatching of eggs

Artificial fertilizations were conducted in November 2004 using Arctic charr from parental stocks maintained in Enonkoski Research Station by Saimaa Aquaculture and Fisheries Research (Finnish Fisheries and Game Research Institute; now renamed as Natural Resources Institute Finland) for captive breeding of the critically endangered Lake Saimaa population. We used milt from 20 males and eggs from 60 females to produce 60 individual families. Eggs from three females were always fertilized with milt of one male, resulting in 20 independent fertilization “blocks” of one male and three females.

Fertilizations were conducted in 1‐L plastic bowls using routine Finnish Game and Fisheries Research Institute procedures. Eggs stripped from each three females (within each 20 block) were fertilized with freshly stripped milt from one male. After 10 min, eggs were flushed in hatchery tanks, and eggs within each fertilization block were divided into four separate cubes on hatching arrays with eight cubes (ca. 200 eggs in each) to produce four replicates. Hatching arrays were randomly placed into four hatchery troughs with a constant water flow (3 L/min). The distance of the family replicates from the water inflow was standardized to remove any block effects from different oxygen concentrations and physical disturbance levels caused by water flow differences inside the troughs. Because of the practical space limitations and varying egg mortality, we were only able to rear families from 11 randomly selected sires (using two to three random dams) with only two randomly selected replicates, in total 56 rearing units.

### 2.2 Rearing of juvenile fish

At the end of the behavioral trials, the fish were of approximately 8.5 months of postfertilization age. The families were reared in plastic boxes (72.5 cm length × 34.5 cm width × 18.0 cm height) with constant water flow (ca. 6 L/min). Until 22 June 2005, water came only from Lake Pahkajärvi (water from 7 m depth, 10–11°C). After 22 June 2005, a combination of Lake Pahkajärvi and Lake Ylä‐Enonvesi water was used to obtain higher temperatures (12–15°C) during the experiment. The combination of two different lake waters was used to maintain sufficiently high level of oxygen dissolved in the water. Half‐sib families (two to three full‐sib families sired by the same male) were randomly placed in the hatchery, and replicate groups were reared in the boxes. Artificial lighting (7:00 to 20:00) was provided in addition to natural light coming through small windows of the hatchery building. Fish were fed on a commercial salmonid food pellets, provided ad libitum with a 16‐hr daily feeding schedule. Rearing boxes were cleaned twice a week.

### 2.3 Antipredator trials

Antipredator trials started on 29 June and lasted until 27 July 2005. Size of fish used in the trials was (mean length ± *SD*) 39.1 ± 3.3 mm (*n* = 557). We observed the behavior of ca. 20 charr from each family (ca. 10 fish from each replicate). The studied fish were exposed to burbot (*Lota lota* L.) odor (odor of two burbots fasted for at least 5 days prior to the experiments; see Vilhunen, Tiira, Laurila, & Hirvonen, 2008 for detailed methods).

The charr used in the behavioral observations were placed into a small rectangular acclimation aquarium (40 cm length × 25 cm width × 42 cm height) approximately 12 hr before the experiment, and then fed by hand. No food was provided to fish during the observation day. Individual charr were gently caught with a net and transferred into our experimental arena, a two‐choice fluviarium (122 length × 35 width × 12 cm height; for details and figure, see Vilhunen, 2006), avoiding unnecessary chasing to minimize handling stress. At this point, the fluviarium contained a 10‐cm layer of fresh, nonburbot odored, lake water, but no waterflow. Each fish was allowed to calm down in the chamber of the fluviarium for 10 min. After a calming period, water inflow was set (one randomly selected side for burbot odor water and another side for fresh water). The mesh gates were opened and a trial started. Mean water flow close to the water source in both channels was 1.3 cm/s at the surface and 3 cm/s at the bottom. We observed the behavior of individual charr for 10 min, during which several behavioral traits were recorded (see below). After each observation, the fluviarium was washed with 80% ethanol and carefully rinsed with lake water to remove all scents. Observed charr were killed with overdose concentration (400 mg/L) of MS‐222 (tricaine methanesulfonate), and their lengths and weights were measured.

Spatial avoidance of burbot odors was examined by recording (i) the proportion of time spent in the scented channel of the total time spent in the channels (avoidance vs. boldness), and (ii) relative proportion of direct approaches of all approaches to the scented channel, indicating boldness of charr to approach the predator. Moreover, (iii) startled responses (sudden and fast swimming bursts) were counted as indicators of escape behavior. As prey fish, including Arctic charr juveniles, are known to respond to the presence of predator by reducing their activity (e.g., Vainikka, Jokelainen, Kortet, & Ylönen, 2005a; Vilhunen & Hirvonen, 2003), we also recorded (iv) total freezing time (time when fish was totally immobile, i.e., frozen), (v) number of freezes, and (vi) relative swimming activity (number of crossed lines drawn on the bottom of the fluviarium in relation to total time spent moving). Indications for heritable sire‐level variation were found in direct approaches to the predator channel, in number of freezes, and in relative swimming activity, but not in proportion of time spent in the predator channel or in startle responses (T. Lautala, unpublished data). Thus, here we studied mean sire‐level correlations over dams between parasite load, relative spleen size and only those above‐mentioned behavioral traits that showed possible indications for heritable variation.

### 2.4 Spleen size and parasite load

Due to practical limitations, we were not able to study parasitism from the same individuals that were studied for behaviors. Thus, 3 weeks after the last behavioral trials, a separate sample of ca. 20 fish per family (ca. 10 fish each replicate) was taken to measure relative spleen size and *Diplostomum* spp. eye‐fluke infestations. Size of the studied fish was (mean length ± *SD*) 52.8 ± 6.6 mm (*n* = 545). The fish were anesthetized, killed with a sharp blow to the head, and dissected for collection of the spleen. Spleen was weighted with the 0.0001 g accuracy microbalance. The spleen is an important antibody producing organ in teleosts (Manning, 1994), and its relative size has been used as a measure of immune function in fish studies (e.g., Kortet, Taskinen, Sinisalo, & Jokinen, 2003; Skarstein & Folstad, 1996; Vainikka et al., 2005b).

Infection with *Diplostomum* spp. parasites occurred naturally with free swimming cercariae from the incoming lake water. The exposure of the parasites was equal for every family, as the water flow was adjusted to be the same for each randomized rearing box. Vulnerability to *Diplostomum* spp. was studied by squashing the lens and vitreous body between two glass plates followed by microscopic examinations. Only four *Diplostomum* spp. individuals and additional two *Tylodelphys* sp. individuals were found from the vitreous body. Thus, these diminutively numbered eye flukes from the vitreous body were excluded from the study, and the further analyzes were performed on more numerous eye flukes from the lens. We consider all the eye flukes located in the lens as *Diplostomum spathaceum*. However, as the taxonomy of *Diplostomum* spp. is problematic and not completely resolved (Valtonen & Gibson, 1997), we recognize the possibility that another related species, *D. pseudospathaceum*, might also have been infecting the study fish (e.g., Seppälä, Louhi, Karvonen, Rellstab, & Jokela, 2015). The life cycle of the *Diplostomum* spp. includes three hosts: a freshwater snail (*Lymnea* and *Radix* spp.), a fish (several species), and a fish‐eating bird (several species) where sexual reproduction occurs. Free swimming cercariae produced in snails infect fish by mostly entering the body through the gills (Mikheev, Pasternak, Valtonen, & Taskinen, 2014) and by migrating to the lens of the eye where they form a long‐lived metacercarial stage.

Low counts of eye flukes in fish eyes indirectly indicate the ability to resist infections, as the fish immune system may respond to these parasites before they reach the eyes (Chappel, 1995). Results by Voutilainen, Huuskonen, and Taskinen (2010) indicate that less than half of *Diplostomum* cercariae that penetrate the charr enter the eye, suggesting the key role of the immune defense of fish. In addition to immunological response against *Diplostomum* parasites, there is indication that fish could behaviorally avoid free swimming cercariae in wild. However, when there is no escape from cercariae, like in our rearing boxes, this type of the avoiding behavior is not effective (Karvonen, Seppälä, & Valtonen, 2004b).

### 2.5 Statistics

The effect of fertilization blocks (“sires”), and dams (nested within sires) (random factors) on parasite load (parasite numbers) and on spleen size (log transformed), was studied using generalized linear mixed model (GLMM, with poisson error distribution and log link function) and linear mixed model (LMM), respectively. Both models also included fish length as a fixed covariate. GLMM was used since distribution of the parasite variables could not be normalized with transformations. For the sire‐level correlations between immune variables and behavioral traits in antipredation, we used average values of traits for sires that were calculated over dams within each fertilization block. To control for size dependency of the studied variables in the correlation analyzes, we used residuals that were extracted from a linear regression of the variables on length or weight (i.e., the relative values that are independent from fish size) (Maxwell & Delaney, 1990). LMM and GLMM analyzes were conducted using lme4 (version 1.1–12) and glmmADMB (version 0.8.3.3) packages (respectively) in R (version 3.2.3). All other analyzes were performed using SPSS (version 21.0.0).

## 3 RESULTS

**Figure 1:**
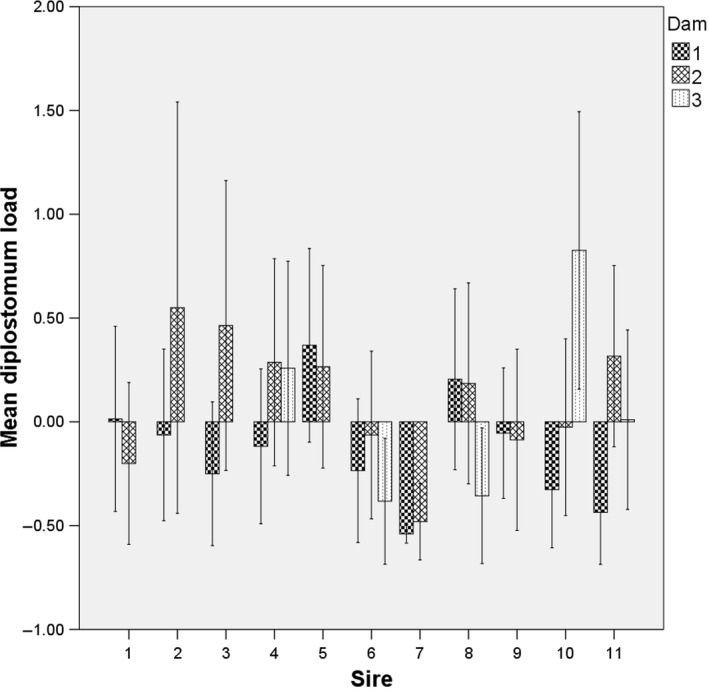
Mean *Diplostomum* parasite loads (length adjusted standardized residuals) for the dams nested under the sires (mean ± *SE*)

Over the family groups, fish length was positively correlated with both the parasite load (*n* = 545, Spearman two‐tailed *r* = .146, *p* < .001), and spleen size (*n* = 545, Spearman two‐tailed *r* = .667, *p* < .001). The mean sire‐level values over dams within each fertilization block for the studied behavioral responses are given in Table 1. All the sires produced families that were parasitized with *Diplostomum* parasites, average loads varying from 0 to 0.95 parasites per fish in a half‐sib group (Figure 1). The spleen size varied from 0.0001 to 0.0050 g, mean value being 0.0009 ± 0.0006 g (*SD*). However, the length‐adjusted spleen size was not significantly correlated to length‐adjusted *Diplostomum* parasite load in mean sire level over dams (*n* = 11, Pearson two‐tailed *r* = −.273, *p* = .417).

**Table 1:** Mean sire‐level values over dams ± one standard error for the studied behavioral responses for the juvenile Arctic charr

Behavioral trait	
Time spent in predator channel (proportion)	0.44 ± 0.01 *n *=* *11
Direct approaches toward predator (proportion)	0.32 ± 0.03 *n *=* *11
Startle responses	0.52 ± 0.06 *n *=* *11
Number of freezes	0.99 ± 0.15 *n *=* *11
Relative swimming activity	0.17 ± 0.01 *n *=* *11

Total parasite load was affected by fish length (GLMM, Z = 3.82, *p* < .001) and dam (likelihood ratio test: *p* < .001), but no difference was found between fertilization blocks (likelihood ratio test: *p* = 1.0). Spleen size was not affected by dams (LMM, χ^2^ = 0.73, *p* = .44, *df* = 1, 1.5% total variance explained) nor fertilization block (χ^2^ = 0.00, *p* = 1.0, *df* = 1, 0% total variance explained) but was affected by fish length (*t* = 22.01, *p* < .001).

The correlations between parasite load, relative spleen size, and those behavioral traits that were known to show heritability are given in Table 2. The results suggest that the fish from the families suffering higher loads by eye flukes were behaving more actively in the predator trials than those from the low parasite load families. Other correlations between behavioral traits and parasite load, and all the correlations between spleen size and behavioral traits were nonsignificant (Table 2).

**Table 2:** Two‐tailed Pearson correlations in the mean sire level over dams between parasitism and immune defense and behavioral traits that showed heritable variation

Behavioral trait	Diplostomum spp. parasite load	Relative spleen size
Direct approaches toward predator	0.496 *n *=* *11, *p *=* *.121	−0.176 *n *=* *11, *p *=* *.824
Number of freezes	−0.318 *n *=* *11, *p *=* *.340	−0.168 *n *=* *11, *p *=* *.622
Relative swimming activity	0.606 *n *=* *11, *p *=* *.048	0.188*n *=* *11, *p *=* *.580

See material and methods for the details of the studied variables.

## 4 DISCUSSION

Our results demonstrate that there are likely maternally derived differences in vulnerability to *Diplostomum* spp. eye‐fluke parasites among Arctic charr individuals, and that vulnerability to eye‐fluke parasitism and behavioral activity in the presence of predator might be associated with each other at mean sire level over dams. As we used fish from a hatchery background population, the current results could suggest that a trade‐off between antipredator behavior and immunity should be considered as a potential contributor to unwanted hatchery effects.

Our data indicate the existence of a clear maternal effect in vulnerability to *Diplostomum* parasitism among juvenile charr is in line with the earlier results on the heritability of *Diplostomum* associated cataract formation in rainbow trout *Oncorhynchus mykiss* (Kuukka‐Anttila et al., 2010). Some of the sires in charr seem to produce notably more resistant offspring against *Diplostomum* parasites with one dam than the other dam combination (Figure 1). Thus, some of the sire‐dam combinations may potentially be a better match to each other than the other combinations—a pattern that was found earlier in offspring resistance against bacterial infections in the whitefish (Wedekind & Müller, 2004). In wild Arctic charr, the prevalence and intensities of *Diplostomum* parasites can be notably high, suggesting high potential for natural selection to work (e.g., Skarstein, Folstad, & Rønning, 2005). Thus, it may be possible that in fish farms, artificial fertilization practices could produce offspring that poorly resists *Diplostomum* parasites. *Diplostomum* infections, on the other hand, are known to increase the risk of predation (Gopko et al., 2017; Mikheev et al., 2010; Seppälä et al., 2004). This could result in the poor survival of these fish in the wild after release. The current number of released hatchery‐raised Arctic charr in critically endangered Lake Saimaa population surviving to the age of the maturity has been notably low (discussion in Vilhunen & Hirvonen, 2003; Vilhunen et al., 2008). The potentially increased risk of predation caused by increased vulnerability to *Diplostomum* parasites may be one of the key factors for their poor survival, and further studies are needed.

Hatchery‐reared fish populations might have unintentionally been selected to favor certain behavioral traits, like abnormal feeding activity or boldness in antipredation response (e.g., Álvarez & Nicieza, 2003; Huntingford, 2004; Olla et al., 1998; Thorpe, 2004). Recent research indicates that salmonids show heritable components also in their behavioral traits (e.g., Kortet, Vainikka, Janhunen, Piironen, & Hyvärinen, 2014). *Diplostomum* spp. parasites have been demonstrated to cause increased risk for avian predation (Seppälä et al., 2004). Thus, in the wild, heavily parasitized individuals are less likely to survive to the age of maturity than less parasitized fish. However, it is presumable that in fish farms with no predation, the individuals with weaker investment in resistance against *Diplostomum* are likely contributing in the gene pool more often than they would do in the wild. If the vulnerability to *Diplostomum* parasites is traded off with other traits, like activity, that are potentially beneficial in the fish farm environment, we would predict a positive sire‐level correlation between parasite load and this type of trait. This prediction is in line with our present findings, suggesting that the sire‐level families behaving bolder (more actively) in the predator trials suffered higher loads by eye flukes than those from less bold‐behaving sire‐level families. Unfortunately, due to practical limitations, we were not able to study parasitism from the same individuals that were studied for their behaviors, which should be tackled in the future work.

The positive sire‐level association between behavioral activity and the load of *Diplostomum* parasites could be due to an energetic trade‐off. Immunologic responses are generally energy demanding (Lochmiller & Deerenberg, 2000) and responses against *Diplostomum* include at least a respiratory burst reaction, that is the production of reactive oxygen species by granulocytes and macrophages (e.g., Kalbe & Kurtz, 2006). Based on our preliminary pilot observations, it seems that those juvenile Arctic charr that swim actively in antipredation trials, do so also when the predator is not present. Thus, it is likely that the energy used for active swimming could incur a cost for the immunologic resistance that results in the current observation. Alternatively, a fish that swims actively has higher exposure to the parasites. However, active swimming should help the fish to escape the parasite cercariae, as suggested by Karvonen et al. (2004b), rather than increase the exposure to *Diplostomum*. Moreover, the exposure by parasites in our study was controlled. Interestingly, Mikheev et al. (2014) demonstrated in the rainbow trout that increased ventilation can increase risk of *Diplostomum* eye‐fluke parasitism, as the main penetration into fish body took place via gills. This could provide a direct link between swimming and infection; higher swimming activity increases ventilation which increases exposure to *Diplostomum* cercariae. This proximate mechanism is well in the line with our findings as increased activity would likely be associated with increased ventilation. Nevertheless, if we think about the survival of these fish after their release to nature, it is important to note that their boldness under predation risk was coupled with increased vulnerability to the eye flukes. However, as correlation was statistically borderline significant, this result should be treated with special caution.

The first infective cercariae of *Diplostomum* are found in waters in Finland usually in early June. Thus, it is likely that some of the fish studied in antipredatory trials have been carrying metacercariae of *Diplostomum* while being tested, but the age of the metacercariae during the behavioral tests (between late June and late July) was almost invariably less than 40 days which is required for the maturation of *Diplostomum* metacercariae (Sweeting, 1974). Before *Diplostomum* larvae are fully developed, they modify host behavior to suppress predation risk (Gopko et al., 2015; Mikheev et al., 2010). Therefore, possible *Diplostomum* infections should not affect the interpretation of our finding about positive sire‐level association between parasite load and behavioral activity. Thus, in our experiment, the possible *Diplostomum* metacercariae would have reduced, rather than increased, the swimming activity of fish near a piscivore predator (see Gopko et al., 2015). Gopko et al. (2017) found in rainbow trout that fish harboring fully developed and mature *Diplostomum* metacercariae increased their activity, preferred to stay closer to the water surface and spent less time immobile after the simulated avian predator attack compared to the control fish. In addition, immature *Diplostomum* can decrease aggressiveness of fish, suppressing predation risk, while hosts with mature parasites behave more aggressively despite this loose contest for territory against uninfected fish, enhancing predation risk (Mikheev et al., 2010).

The family background did not have any effect on relative spleen size. Relative spleen size was neither significantly correlated to length‐adjusted *Diplostomum* parasite load. The spleen is involved in haematopoiesis, antibody production and in clearance of foreign particles, pathogens, and moribund cells from the blood stream (Dalmo, Ingebritsen, & Bøgwald, 1997; Manning, 1994). A proportionally large spleen is thought to be a good measure of condition and the ability to respond to infection (Seppänen, Kuukka, Voutilainen, Huuskonen, & Peuhkuri, 2009; Wester, Vethaak, & van Muiswinkelm, 1994). The fish immune system is suggested to respond to free swimming *Diplostomum* cercariae in the tissues before the cercariae reach the eyes (Chappel, 1995). Our results contrast the findings by Seppänen et al. (2009), who reported significant spleen enlargement in *Diplostomum* infected juvenile Arctic charr. Therefore, it seems that the relative size of the spleen cannot act as an unambiguous indicator of the resistance against *Diplostomum* cercariae. Supporting this view, in wild tench, *Tinca tinca*, Vainikka, Kortet, Paukku, Rantala, and Pirhonen (2005c) failed to find any association between *Diplostomum* eye‐fluke load and relative size of the spleen.

To conclude, in the present fish stock, held in hatchery environment for two generations, there were maternally derived differences in vulnerability to Diplostomum eye flukes, and the presumably bold performance under predation risk at the sire level was coupled with increased vulnerability to the eye flukes. The future studies should tackle these topics in more detail to increase our understanding of the problems associated with fish farm stock populations used in restocking programs.

## CONFLICT OF INTEREST

Authors do not have any conflict of interests.

## AUTHORS CONTRIBUTION

HH and RK produced the original idea and experimental design of the study. RK, TL, and JT collected the data. JK participated data analysis and writing, together with the other co‐authors.
